# External validation of the LENT and PROMISE prognostic scores for malignant pleural effusion

**DOI:** 10.1183/23120541.01019-2024

**Published:** 2025-06-16

**Authors:** Craig A. Mounsey, Nikolaos I. Kanellakis, Dinesh N. Addala, Jamie A. Mawhinney, Nick Freemantle, Najib M. Rahman

**Affiliations:** 1 Oxford Pleural Unit, Oxford University Hospitals, Oxford, UK; 2 Chinese Academy of Medical Science Oxford Institute, Nuffield Department of Medicine, Medical Sciences Division, Oxford University, Oxford, UK; 3 Oxford Respiratory Trials Unit, Nuffield Department of Medicine, University of Oxford, Oxford, UK; 4 Portsmouth Hospitals University NHS Trust, Portsmouth, UK; 5 Institute of Clinical Trials and Methodology, University College, London, UK

## Abstract

**Background:**

Accurate survival estimation in malignant pleural effusion is essential to guide clinical management strategies and inform patient discussion. The LENT and PROMISE scores were developed to aid prognostication in malignant pleural effusion; however their uptake in practice has been limited. We aimed to conduct a detailed external validation of the LENT and PROMISE scores to develop recommendations regarding clinical utility, and to highlight factors limiting performance.

**Methods:**

Medical records of patients diagnosed with malignant pleural effusion between 2015–2023 at Oxford University Hospitals were retrospectively reviewed to determine length of survival and the LENT and PROMISE scores at diagnosis. Performance of the scores in predicting overall survival and chance of survival at 3, 6 and 12 months was assessed using measures of discrimination, calibration and overall model performance. Kaplan–Meier analysis and Cox models were utilised to further investigate individual score variables.

**Results:**

773 patients with malignant pleural effusion were included. Both scores showed predictive ability for overall survival; however median survival estimates lacked precision. Score performance in predicting survival at 3, 6 and 12 months was stronger, with C-indices around 0.8 for both at each time point, and the models appearing well calibrated. Limited stratification of tumour types and lack of consideration of sensitising mutations were demonstrated to be potential factors restricting performance.

**Conclusions:**

Both scores have the ability to prognosticate in malignant pleural effusion, and greater use in practice should be considered. However, areas to improve score performance were also highlighted, and these may aid future model development.

## Shareable abstract

**The LENT and PROMISE scores have strong predictive ability for 3-, 6- and 12-month mortality in malignant pleural effusion. Accurate prognostication is vital for patient management and hence greater use of the scores in practice should be considered.** https://bit.ly/3Oj2lVq

## Introduction

Malignant pleural effusion (MPE) is a common clinical condition affecting ∼15% of patients with cancer [1, 2]. The current incidence of MPE in the UK is estimated to be 50 000 individuals per year [3], and, with the growing rate of new cancer diagnoses, this looks set to increase [4, 5].

The presence of MPE indicates advanced disease, and median survival is typically quoted as 3–12 months [1, 6]. However, survival time can be extremely variable between individuals with some surviving many years from MPE diagnosis [7, 8]. Accurate survival prediction in this cohort is essential and significantly impacts clinical management, with a need to balance likely survival time against the frequency of hospital visits or admission for fluid control procedures [9]. International guidelines suggest that those with longer survival should undergo definite interventions such as talc pleurodesis or indwelling pleural catheter (IPC) insertion, while a pragmatic approach (such as repeat thoracocentesis) to minimise healthcare contact should be prioritised for those with shorter survival [1, 10]. Furthermore, prognostic uncertainty has been demonstrated to cause significant distress in patients with advanced cancer [11], and therefore the provision of accurate survival information may improve quality of life in those diagnosed with MPE.

Physicians are notoriously poor at approximating prognosis in patients with this condition [9]. This has been demonstrated in large-scale clinical trials such as TIME-2, where, despite an exclusion criterion being an estimated survival of <3 months, 34% of participants died within this timeframe [12]. To address this, a number of scores aiming to predict survival in patients with MPE have been developed and validated, the two most prominent being the LENT [13] and PROMISE [14] scores. The LENT score (table 1) was developed from three prospectively collected databases from the UK, Australia and the Netherlands, and categorises patients into low-, moderate- or high-risk groups with estimated median survival times of 319, 130 and 44 days, respectively. The PROMISE score (table 2) was developed to predict 90-day mortality using data from the TIME-1 [15], TIME-2 [12] and TIME-3 [16] multicentre clinical trials. The PROMISE score categorises patients into four risk groups, ranging from Group A (<25% 90-day mortality) to Group D (≥75% 90-day mortality).

**TABLE 1:** LENT score calculation and risk categories

Variable	Score
**Pleural fluid LDH U·L^−1^**	
** **<1500	0
** **>1500	1
**ECOG PS**	
** **0	0
** **1	1
** **2	2
** **3–4	3
**NLR**	
** **<9	0
** **>9	1
**Tumour type**	
** **Mesothelioma or haematological	0
** **Breast, gynaecological or RCC	1
** **Lung or other	2

Risk category (score)	Estimated median survival days
**Low risk (0–1)**	319
**Moderate risk (2–4)**	130
**High risk (5–7)**	44

LDH: lactate dehydrogenase; ECOG PS: Eastern Cooperative Oncology Group Performance Status; NLR: neutrophil-to-lymphocyte ratio; RCC: renal cell carcinoma.

**TABLE 2:** Clinical PROMISE score calculation and risk categories

Variable	Score
**Previous chemotherapy**	
** **No	0
** **Yes	4
**Previous radiotherapy**	
** **No	0
** **Yes	2
**Haemoglobin g·dL^−1^**	
** **>16	0
** **14 to <16	1
** **12 to <14	2
** **10 to <12	3
** **<10	4
**White cell count 10⁹ cells·L^−1^**	
** **<4	0
** **4 to <6.3	2
** **6.3 to <10	4
** **10 to <15.8	7
** **>15.8	10
**C-reactive protein IU·L^−1^**	
** **<3	0
** **3 to <10	3
** **10 to <32	5
** **32 to <100	8
** **>100	11
**ECOG PS**	
** **0–1	0
** **2–4	7
**Tumour type**	
** **Mesothelioma	0
** **Other	4
** **Lung	5

Risk category (score)	3-month mortality risk %
**A (0–20)**	<25
**B (21–27)**	25 to <50
**C (28–35)**	50 to <75
**D (>35)**	≥75

ECOG PS: Eastern Cooperative Oncology Group Performance Status.

However, these prediction models have failed to be widely embraced in clinical practice [9]. This is due, at least in part, to a lack of thorough external validation since their production. In addition, it has been suggested that the predictive ability of the scores may be limited by the variables included and the nature of their use. For instance, the categorisation of tumour types in both scores into three risk groups has been argued to insufficiently reflect the heterogeneity between different underlying malignancies [17, 18], while the lack of inclusion of sensitising mutations fails to account for molecular targeted therapies that may alter survival expectations [19–21].

In the present study, we evaluated the performance of the LENT and PROMISE scores in the largest independent cohort to date, allowing a modern assessment of clinical utility and therefore recommendations to be made regarding their use in practice. Both overall survival and the chance of survival at 3, 6 and 12 months from MPE diagnosis were assessed to allow consideration of the optimal output from the scores. In addition, we utilised our analysis to highlight potential areas limiting the predictive capabilities of the scores, and suggest possible improvements for the development of future MPE prognostic models.

## Methods

### Ethics

Ethical and regulatory approval for the study was obtained through Health Research Authority (HRA) and Health and Care Research Wales (HCRW) Approval (REC reference number 24/HRA/1980).

### Participants and eligibility

Patients with MPE were identified through retrospective analysis of the Oxford Pleural Database. This is a comprehensive record of all patients discussed at the Oxford University Hospitals NHS Foundation Trust (OUH) pleural multidisciplinary team (MDT) meeting since July 2015 (n=2568). The OUH pleural MDT is a weekly meeting in which all patients managed by the pleural team at OUH are discussed on a case-by-case basis. For the current study, patients reviewed at the OUH pleural MDT between July 2015 and October 2023 with a final diagnosis of MPE had their electronic medical records screened. Patients were only excluded in the presence of key missing data, specifically insufficient baseline information to determine date of MPE diagnosis or insufficient follow-up information regarding length of survival. Patients were included in the absence of confirmatory pleural fluid cytology or pleural histopathology if a diagnosis of MPE was made following pleural MDT discussion. In these cases the diagnosis was based on radiological and clinical features, such as a large effusion with radiological characteristics suggestive of malignancy in the presence of known metastatic cancer.

### Data collection

Data were collected from the electronic patient records of identified individuals in April 2024, which was at least 6 months after the date of MPE diagnosis for all patients. Data collected included age, sex, date of MPE diagnosis, date of death (if applicable), causative cancer type, pleural fluid lactate dehydrogenase (LDH), Eastern Cooperative Oncology Group Performance Status (ECOG PS), previous chemotherapy and radiotherapy, and baseline blood results, including neutrophils, lymphocytes, C-reactive protein (CRP), haemoglobin (Hb) and white cell count (WCC). For patients with lung adenocarcinoma, the presence or absence of sensitising mutations (EGFR, ALK, ROS and BRAF) was recorded. Survival was calculated as the time from MPE diagnosis to the date of death. Patients were censored at the time of data collection or loss to follow-up. The LENT [13] and clinical PROMISE [14] scores at diagnosis were calculated and risk categories determined.

### Statistical analysis

Descriptive statistics were expressed as frequency (percentages) for categorical variables and median (interquartile range (IQR)) for continuous variables. Kaplan–Meier survival curves were used to compare cumulative survival rates and log-rank (Mantel–Cox) tests were used to assess for differences between groups. For model discrimination, Cox regression was performed to compare relative survival, while receiver operating characteristic (ROC) curves and area under the ROC (AUROC) curves were used to calculate the C-statistic for mortality at specified timepoints. For model calibration, plots of the predicted *versus* observed mortality were produced, and the calibration slopes were calculated. The Brier and Nagelkerke's R^2^ scores were calculated as general measures of model overall performance. All statistical tests were two-sided and statistical significance was set at p<0.05. Missing data were handled through complete-case analysis for all tests. Statistical analyses were performed using IBM SPSS Statistics (Version 29.0.1.0), R (version 4.4.0; www.R-project.org/) and SAS V 9.4 (SAS Institute; Cary, NC, USA).

### Study reporting

The study was reported following the Transparent Reporting of a multivariable prediction model for Individual Prognosis Or Diagnosis (TRIPOD) statement [22].

## Results

### Cohort demographics and clinical characteristics

A total of 812 patients with MPE were identified. Of these, 39 (4.8%) had either inadequate baseline or follow-up information and were excluded. 390 of the remaining patients were female (50.5%), and 383 were male (49.5%) (table 3). The median survival from MPE diagnosis was 158 days (IQR 58–533), and the median age at MPE diagnosis was 72 years (IQR 63–80). This was similar to the median age of the PROMISE development cohort (71 years), but marginally greater than that of the LENT score (mean 66 years). Causative malignancies in our cohort were also similar to the LENT and PROMISE development cohorts, with common cancer types including lung (28.6%), mesothelioma (21.1%), breast (16.9%), and malignancy of gastrointestinal (9.3%) or gynaecological origin (9.2%). In the majority of included patients the diagnosis was confirmed through cytology and/or histopathology (89.1%). The remaining patients were diagnosed with MPE based on radiological and clinical features following discussion at MDT. At the time of analysis, 681 (88.1%) of the included patients had died while 92 remained alive. Sufficient data were present to allow LENT score calculation in 699 patients (90.4%) and PROMISE score calculation in 636 patients (82.3%). Both scores could be calculated in 602 patients (77.9%). Median follow-up duration in our cohort was 157 days.

**TABLE 3:** Patient demographics and clinical characteristics

**Age years**	72 (63–80)
**Sex**	
Female	390 (50.5)
Male	383 (49.5)
**Primary cancer**	
Lung	221 (28.6)
Adenocarcinoma	177
Small cell	21
Squamous cell	15
Unspecified	8
Mesothelioma	163 (21.1)
Epithelioid	115
Biphasic	26
Sarcomatoid	17
Unspecified	5
Breast	131 (16.9)
Gastrointestinal	72 (9.3)
Pancreatic	23
Oesophageal	22
Colorectal	15
Cholangiocarcinoma	7
Gastric	5
Gynaecological	71 (9.2)
Haematological	33 (4.3)
Sarcoma	17 (2.2)
Renal cell	16 (2.1)
**ECOG PS**	
0	134 (17.7)
1	296 (39.0)
2	175 (23.1)
3–4	154 (20.3)
**LENT category**	
Low	125 (17.9)
Moderate	437 (62.5)
High	137 (19.6)
**PROMISE category**	
A	272 (42.8)
B	190 (29.9)
C	153 (24.1)
D	21 (3.3)
**Previous chemotherapy**	
Yes	183 (30.4)
No	419 (69.6)
**Previous radiotherapy**	
Yes	148 (24.6)
No	454 (75.4)

Data are presented as n (%) or median (IQR). ECOG PS: Eastern Cooperative Oncology Group Performance Status.

### LENT and PROMISE score performance for overall survival

**FIGURE 1:**
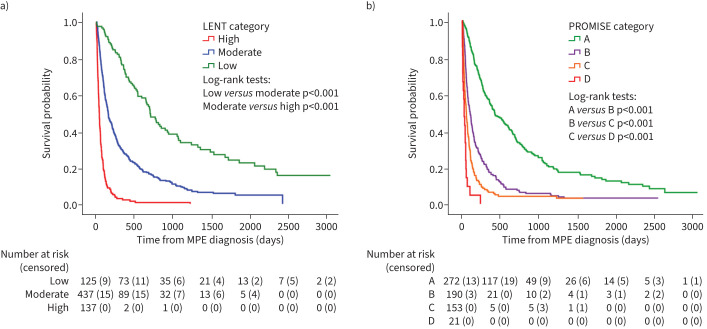
Kaplan–Meier survival curves for patients stratified by a) LENT and b) clinical PROMISE score categories. MPE: malignant pleural effusion.

The median (IQR) lengths of survival for patients in the low-, moderate- and high-risk LENT categories were 705 (360–1728), 158 (70–429) and 42 days (20–79), respectively, while in PROMISE categories A–D they were 424 (192–1040), 104 (48–249), 57 (25–112) and 28 days (10–48). Survival experience by LENT and PROMISE categories is demonstrated in figure 1. Harrell's C-statistic was 0.72 (95% CI 0.70–0.74) and 0.73 (95% CI 0.71–0.75) for the LENT and PROMISE scores, respectively.

### LENT and PROMISE score performance for 3-, 6- and 12-month mortality

**FIGURE 2:**
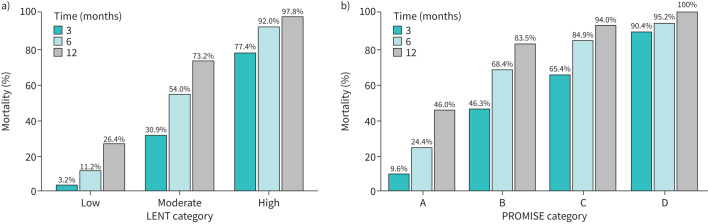
Proportion of patients surviving at each timepoint for a) LENT and b) clinical PROMISE score categories.

**FIGURE 3:**
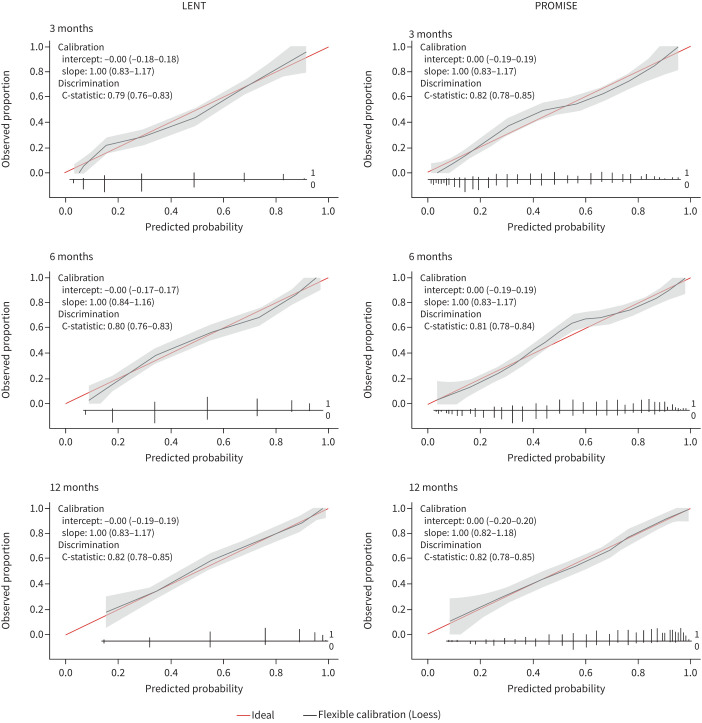
Calibration plots for the LENT and PROMISE scores at 3, 6 and 12 months.

In addition to assessing likely overall survival, prognostic scores can also be utilised to predict the chance of patient survival at subsequent timepoints. We therefore assessed the ability of the LENT and PROMISE scores to predict survival at 3, 6 and 12 months from MPE diagnosis. The proportion of patients in each LENT and PROMISE score category surviving at these timepoints is shown in figure 2. Of note, the 3-month mortality proportions in this cohort were consistent with the outcomes predicted by the PROMISE score output [14]. Performance characteristics of the LENT and PROMISE scores at each timepoint are shown in table 4. The Akaike information criterion values, Brier scores and Nagelkerke's R^2^ scores were calculated from patients in whom both scores were available to allow comparison, and, in general, the performance characteristics of the PROMISE score were marginally superior. Calibration plots for each time point are shown in figure 3.

**TABLE 4:** Performance characteristics of the LENT and clinical PROMISE scores for 3-, 6- and 12-month mortality

	3-month mortality	6-month mortality	12-month mortality
**LENT score**			
C-statistic (95% CI)	0.79 (0.76–0.83)	0.80 (0.76–0.83)	0.82 (0.78–0.85)
Calibration slope (95% CI)	1.00 (0.83–1.17)	1.00 (0.84–1.16)	1.00 (0.83–1.17)
Brier score	0.175	0.176	0.149
Nagelkerke's R^2^	0.325	0.372	0.366
AIC	632.8	634.2	540.2
**PROMISE score**			
C-statistic (95% CI)	0.82 (0.78–0.85)	0.81 (0.78–0.84)	0.82 (0.78–0.85)
Calibration slope (95% CI)	1.00 (0.83–1.17)	1.00 (0.83–1.17)	1.00 (0.82–1.18)
Brier score	0.165	0.174	0.150
Nagelkerke's R^2^	0.388	0.379	0.352
AIC	595.2	629.3	548.1

AIC: Akaike information criterion.

### Evaluating the performance of variables used in the LENT and PROMISE scores

#### Cox regression

To evaluate the performance of the individual LENT and PROMISE score variables, univariate Cox regression models were constructed with each variable categorised as per their use in either the LENT or PROMISE score. With the exception of previous radiotherapy, all of the variables used in the scores were significantly associated with survival (table 5). Multivariable Cox regression analyses with the combination of variables used in either the LENT or PROMISE scores were also conducted, with similar results to univariate analyses (table 5).

**TABLE 5:** Cox regression analyses of survival time on the individual variables used in the LENT and/or PROMISE scores

Variable (ref)	Univariable analysis		Multivariable analysis			
	HR (95% CI)	p-value	HR (95% CI) – LENT variables	p-value	HR (95% CI) – PROMISE variables	p-value
**LDH >1500U·L^−1^**	1.55 (1.20–2.01)	<0.001	1.38 (1.06–1.80)	0.016		
**NLR >9**	2.05 (1.72–2.45)	<0.001	1.52 (1.26–1.85)	<0.001		
**Tumour score (0)**		<0.001		<0.001		<0.001
1	1.30 (1.05–1.61)		1.40 (1.11–1.77)		1.10 (0.84–1.44)	
2	2.67 (2.20–3.25)		2.64 (2.12–3.28)		2.35 (1.86–2.95)	
**ECOG PS (0)**		<0.001		<0.001		<0.001
1	1.74 (1.37–2.21)		1.54 (1.20–1.98)		1.48 (1.13–1.95)	
2	3.05 (2.36–3.95)		2.57 (1.95–3.37)		2.49 (1.84–3.37)	
3	5.85 (4.46–7.66)		4.61 (3.43–6.20)		5.01 (3.62–6.94)	
4	8.66 (4.83–15.52)		9.61 (5.19–17.96)		11.80 (5.89–23.63)	
**Hb g·dL^−1^ (≥16)**		<0.001				0.006
14 to <16	1.10 (0.62–1.93)				1.64 (0.90–3.00)	
12 to <14	1.26 (0.72–2.20)				1.45 (0.80–2.62)	
10 to <12	2.04 (1.16–3.58)				1.71 (0.94–3.11)	
<10	1.91 (1.05–3.47)				0.98 (0.52–1.86)	
**CRP IU·L^−1^ (<3)**		<0.001				<0.001
3 to <10	1.35 (0.92–1.97)				1.09 (0.72–1.64)	
10 to <32	1.91 (1.34–2.73)				1.41 (0.96–2.06)	
32 to <100	2.73 (1.95–3.83)				2.28 (1.56–3.34)	
≥100	4.82 (3.39–6.85)				3.88 (2.57–5.85)	
**WCC 10⁹ cells·L^−1^ (<4)**		<0.001				0.003
4 to <6.3	0.65 (0.38–1.12)				1.03 (0.52–2.02)	
6.3 to <10	0.87 (0.52–1.46)				0.97 (0.51–1.83)	
10 to <15.8	1.02 (0.60–1.73)				0.92 (0.48–1.78)	
≥15.8	1.86 (1.06–3.27)				1.70 (0.86–3.37)	
**Previous chemotherapy**	1.25 (1.06–1.48)	0.008			1.25 (1.00–1.55)	0.049
**Previous radiotherapy**	1.07 (0.90–1.27)	0.46			1.20 (0.95–1.52)	0.13

ref: reference; HR: hazard ratio; LDH: lactate dehydrogenase; NLR: neutrophil–lymphocyte ratio; ECOG PS: Eastern Cooperative Oncology Group Performance Status; Hb: haemoglobin; CRP: C-reactive protein; WCC: white cell count.

#### Further analysis of tumour type

It has been suggested that the stratification of tumour types in the LENT and PROMISE scores does not sufficiently account for the biological variability of different malignancies [17, 18]. To investigate this in our cohort, patients were divided into groups of tumour type as per the LENT score (given that this allowed greater division compared to the PROMISE score), and the median (IQR) lengths of survival were calculated from Kaplan–Meier analysis (table 6). It was notable that survival in those with haematological malignancy appeared markedly better than that in all other cancer types, and this was confirmed through log-rank tests. The survival of patients diagnosed with “other” malignancies was significantly worse than that of those diagnosed with lung cancer (p=0.001).

**TABLE 6:** Median length of survival (LOS) by LENT score malignancy categorisation

Malignancy	Median (IQR) LOS days
**Mesothelioma**	293 (108.5–659.8)
**Haematological**	1326 (350–)
**Breast**	295 (70–1041)
**Gynaecological**	241 (80–549)
**Renal cell carcinoma**	55 (35–214)
**Lung**	112 (44–271)
**Other**	64 (36–140)

In addition to inter-malignancy categorisation, it has also been suggested that consideration of tumour subtypes and sensitising mutations is lacking in the LENT and PROMISE scores [23, 24]. Using mesothelioma as an example, in our cohort the median survival in those with epithelioid mesothelioma was 473 days compared to 182 days in non-epithelioid subtypes (p<0.001). A similar analysis was performed to compare the survival of lung adenocarcinomas to other lung cancer histological subtypes, with no significant difference (p=0.41). We also investigated the impact of sensitising mutations in lung adenocarcinoma patients; 145 patients in our cohort had MPE secondary to lung adenocarcinoma with molecular testing results available. Of these, 28 (19.3%) had a sensitising mutation, most commonly in the EGFR gene (n=22). The median length of survival was 356 days in those with a sensitising mutation compared to 126 days in those without (p<0.005).

## Discussion

To date, the output from the LENT score has predominantly been in the form of median survival time, while the PROMISE score was developed to predict 3-month mortality. In the present study, we assessed the performance of both scores to predict overall survival and the likelihood of survival at the predetermined timepoints of 3, 6 and 12 months.

In terms of overall survival, both scores showed predictive ability, with clear discrimination between the survival curves for their risk categories (figure 1), and a Harrell's C-statistic of 0.72 and 0.73 for the LENT and PROMISE scores, respectively. However, it was notable that while median survival for the moderate- and high-risk LENT categories was similar to those initially reported by Clive *et al*. [13], the median survival of the low-risk category was markedly greater in our cohort (705 days *versus* 319 days). This indicates that the reported LENT score output for low-risk patients may significantly underestimate survival time. Similar findings have been demonstrated in other LENT score validation studies, with Ermin *et al*. [2] finding median survival in their low-risk group to be 27 months, while in the study by Söyler *et al*. [25] this was 31 months. Furthermore, the IQRs for both scores, especially in the lower score categories, were extremely broad, a finding which is also notable from the initial LENT score publication [13]. Therefore, these scores are unlikely to be precise enough to convey meaningful information about “average” survival time to patients outside the highest risk categories. This supports previous arguments from both clinicians and patients that median survival time is not a useful metric for individual patients or clinical decision-making [26, 27].

An alternative form of outcome which can be provided by prognostic scores is the likelihood of survival at subsequent timepoints. The performance of the scores in predicting 3-, 6- and 12-month mortality in our cohort was strong [28], with C-indices around 0.8 for both at each time point, and the models appearing to be well calibrated. These findings are comparable with previous assessments of LENT and PROMISE score performance [18, 23, 29]. Alawneh *et al*. [29] performed a single-centre retrospective external validation of the LENT score in Jordan and showed AUROC curves of 0.74, 0.78 and 0.79 for 1-, 3- and 6-month mortality, respectively. Similarly, Wong *et al*. [23] found AUROC curves of 0.80 for the PROMISE score and 0.77 for the LENT score for 3-month mortality in their evaluation in an Asian population. The chance of survival at predetermined timepoints may therefore be a more useful, accurate and appropriate output for the scores than median survival time. Nonetheless, the form of prognostic information that is most understandable to patients must also be considered, and this may differ between individuals. There is currently a lack of evidence addressing this important issue, and future research to provide greater clarity on the most patient-accessible output from prognostic scores would be beneficial. In terms of the choice between the two scores for predicting survival at predetermined timepoints, the performance characteristics of the PROMISE score were marginally superior in our study (table 4), yet the variables required for LENT score calculation are often more readily available. Therefore, the use of either score would appear to be acceptable.

Given that we have shown that both scores carry predictive ability for survival in MPE, it is important to consider how these models should be used in practice. It is crucial to offer meaningful prognostic information to patients with advanced malignancy to reduce the distress caused by prognostic uncertainty [11], and we would advocate use of the scores for this purpose. The scores may also be useful in determining the appropriateness of patients for inclusion in clinical trials. However, the utility of the scores as an aid to clinical decision-making is currently unclear. While MPE guidelines suggest temporising treatments such as thoracentesis in patients with short life expectancy [1, 10], in practice this is typically only applied to those with extremely poor prognosis, and in remaining individuals the decision surrounding management (*e.g.* talc pleurodesis *versus* IPC insertion) is predominantly led by patient preference rather than options to alter survival [30]. This limited requirement to stratify individuals to different treatment options based on prognosis likely reflects a key reason for the lack of uptake of the scores amongst clinicians. However, it may be that in the near future MPE-specific pharmacological options become available, at which point the clinical utility of prognostic scores will increase. For instance, intrapleural immunotherapeutic [31, 32] and antiangiogenic [33] agents are currently being investigated, with promising initial results.

The aim of the current study was to externally evaluate the performance of the LENT and PROMISE scores, rather than develop a new prognostic model. Nonetheless, our results highlight a number of areas which developers of future MPE prognostic scores should consider. Firstly, we demonstrate through Cox models that, with the exception of previous radiotherapy, each of the variables used in the LENT and PROMISE scores should be assessed for inclusion in future scores. Secondly, we show that the presence of sensitising mutations in the underlying primary malignancy should be considered. Taking lung adenocarcinoma as an example, in this cohort we found that those with sensitising mutations experienced significantly longer survival than those without, likely reflecting the survival benefit introduced by treatment with targeted therapies such as tyrosine kinase inhibitors [21]. This supports previous work demonstrating that in areas with high prevalence of EGFR mutation, the performance of the LENT score can be improved by re-scoring lung adenocarcinomas from the highest to the lowest risk category (EGFR-LENT) [24]. Our results also suggest that further stratification of tumour types may improve model performance. For instance, patients with non-epithelioid mesotheliomas had significantly shorter survival in this cohort than those with an epithelioid subtype, indicating that grouping these may limit predictive ability. In fact, given the heterogeneity in outcomes between malignancies it has previously been suggested that tumour-specific prognostic scores for MPE are required [17, 18]. An example of such an approach is seen with the recently developed Breast and Lung Effusion Survival Score (BLESS) [17], which outperformed the LENT score in the validation cohort but has not been externally validated. Finally, future model creators should consider alternative approaches to the use of continuous variables. In both the LENT and PROMISE scores continuous variables have been categorised (LDH and neutrophil–lymphocyte ratio in the LENT score, CRP, WCC and Hb in the PROMISE score), likely due to a nonlinear relationship between the variables and the outcome. However, this causes substantial information loss from the models and imposes arbitrary cut-offs between categories that have no biological basis [34, 35]. Techniques to allow the use of such variables as continuous have been developed and validated, including cubic splines, and these should be considered in future MPE model development [36, 37].

### Strengths and limitations

A particular strength of this study is the size of patient cohort and the relatively small amount of data loss. To the best of our knowledge, this is the largest cohort to externally assess the performance of the LENT and PROMISE scores to date. In addition, the analysis was conducted using patients diagnosed with MPE over a considerable time period, and until the end of 2023. A key concern with prognostic scores is that their performance may diminish over time with changing healthcare practice and patient demographics [9], yet despite our timeframe including significant advancements in the use of immunotherapy in lung cancer [19] and mesothelioma [20], the performance of the scores appears to remain strong.

Notable limitations include data collection at a single-centre tertiary hospital, which may limit the generalisability of our findings to other healthcare settings, and the retrospective nature of the study. While ideally prospective studies would be performed, due to the relatively low incidence of MPE such studies would take a long time to recruit, and therefore retrospective cohort studies are an appropriate methodology. Also, this evaluation has not considered all prognostic models developed for MPE. Over recent years a number of additional scores have been published, including the tumour-specific BLESS [17] and CAIL [18] scores, and further “one-size-fits-all” scores such as SELECT [21], EGFR-LENT [24] and modified-LENT [38]. We focussed on the LENT and PROMISE scores in this study as the two most prominent models published to date, and to allow inclusion of all tumour types in the analysis. However, these newer scores address some of the limitations with the LENT and PROMISE scores that we have highlighted in this study, and their external validation is therefore certainly warranted.

### Conclusion

In this cohort both the LENT and PROMISE scores demonstrated predictive ability for overall survival, yet the precision of median survival estimates was lacking. The scores are likely to be more useful in determining the chance of survival at specified timepoints, with both scores showing strong performance at the intervals assessed. However, it appears that there is scope for performance of the scores to be improved through greater stratification of tumour types, consideration of sensitising mutations and the inclusion of appropriate variables as continuous. Currently, the utility of the LENT and PROMISE scores will predominantly be in the provision of prognostic information to affected individuals; however as more MPE-specific therapeutic options become available, prognostic scores to stratify patients to different treatments may be required.
